# Clinical and Laboratory Features in the Israeli Population with COVID-19 Infection after Pfizer-BioNTech mRNA Booster Vaccination

**DOI:** 10.3390/vaccines10050636

**Published:** 2022-04-19

**Authors:** Ariel Israel, Eugene Merzon, Yotam Shenhar, Ilan Green, Avivit Golan-Cohen, Alejandro A. Schäffer, Eytan Ruppin, Shlomo Vinker, Eli Magen

**Affiliations:** 1 Leumit Research Institute & Department of Family Medicine, Leumit Health Services, Tel Aviv-Yafo 6473817, Israel; dr.ariel.israel@gmail.com (A.I.); emarzon@leumit.co.il (E.M.); yshenhar@leumit.co.il (Y.S.); igreen@leumit.co.il (I.G.); agolanchoen@leumit.co.il (A.G.-C.); svinker@leumit.co.il (S.V.); 2 The Adelson School of Medicine, Ariel University, Ariel 40700, Israel; 3 Department of Family Medicine, Sackler Faculty of Medicine, Tel-Aviv University, Tel Aviv-Yafo 6997801, Israel; eytan.ruppin@nih.gov; 4 Cancer Data Science Laboratory, National Cancer Institute, Bethesda, MD 20814, USA; alejandro.schaffer@nih.gov; 5 Medicine C Department, Clinical Immunology and Allergy Division, Barzilai University Medical Center, Ben Gurion University of the Negev, Be′er Sheva 8410501, Israel

**Keywords:** BNT162b2, mRNA, vaccine, SARS-CoV-2, infection, convalescents

## Abstract

Background: Immune protection following either vaccination or infection with SARS-CoV-2 decreases over time. Objective: We aim to describe clinical and sociodemographic characteristics associated with COVID-19 infection at least 14 days after booster vaccination in the Israeli population. Methods: We conducted a population-based study among adult members of Leumit Health Services (LHS) in Israel. Nasopharyngeal swabs were examined for SARS-CoV-2 by real-time RT-PCR. The hematological and biochemical parameters in the peripheral blood before booster vaccination were evaluated. Results: Between 1 February 2021 and 30 November 2021, 136,683 individuals in LHS were vaccinated with a booster (third dose) of the BNT162b2 vaccine. Of these, 1171 (0.9%) were diagnosed with COVID-19 by testing positive for SARS-CoV-2 RT-PCR at least >14 days after the booster vaccination. The COVID-19-positive group was characterized by higher rates of chronic kidney disease than the matched COVID-19-negative group (43 (3.7%) vs. 3646 (2.7%); *p* = 0.039). Anemia, lower peripheral blood lymphocytes, monocytes, basophils, C3 Complement, cholesterol, and prothrombin time were also associated with COVID-19 after booster vaccination. Conclusion: People with chronic kidney disease and anemia should be included in possible future annual SARS-CoV-2 vaccination recommendations.

## 1. Introduction

Vaccination against SARS-CoV-2 is an important approach to halting the progression of the COVID-19 pandemic. Israel was among the first countries to initiate a large-scale vaccination campaign, on December 20th 2020, and quickly immunized a high proportion of the adult population, thereby achieving early control over the spread of SARS-CoV-2 [1]. More than five million Israelis (out of 9.3 million) have been fully vaccinated with two doses of the Pfizer-BioNTech vaccine as of 26 May 2021 [2]. National surveillance data from the first four months of Israel’s vaccination campaign revealed that two doses of BNT162b2 (the Pfizer–BioNTech COVID-19 vaccine (tozinameran) reduced both symptomatic and asymptomatic infections, COVID-19-related hospitalizations, severe infection, and mortality [3]. However, our previous large population study, tested for SARS-CoV-2 by RT-PCR after two doses of mRNA BNT162b2 vaccine, has shown a gradual increase in the risk of infection from the time since receiving their second vaccine dose [4]. To clarify this observation, we determined the kinetics of SARS-CoV-2 IgG antibodies following the administration of two doses of BNT162b2 vaccine or with respect to SARS-CoV-2 infection in unvaccinated individuals and found that initial levels of antibodies were much higher in vaccinated patients but decreased faster [5].

The resurgence of COVID-19, caused primarily by the delta variant (B.1.617.2) of SARS-CoV-2, has prompted Israeli authorities to administer a third dose of the mRNA vaccine COVID-19 as a booster dose to counteract possible waning of immunity over time. As a result, the rates of confirmed COVID-19 and severe COVID-19-related outcomes have been significantly reduced in those who received a booster dose of BNT162b2 vaccine [6,7]. However, in recent months, we have been observing new cases of COVID-19 in individuals who have received three doses of the BNT162b2 vaccine. Therefore, identification of individuals who are at an increased risk of infection after their booster vaccination is becoming increasingly important. A previous study of fully vaccinated, predominantly male US veterans (median age 73 years (Interquartile range [IQR] 68–78)) showed that old age and anemia were positively associated with post-vaccination COVID-19 and that blacks were at lower risk than whites [8]. Consequently, this study is not representative of the clinical and sociodemographic risk factors for post-vaccination COVID-19 in the global general population [8].

Therefore, we aimed to describe clinical and sociodemographic characteristics associated with COVID-19 infection at least 14 days after booster vaccination in the Israeli population.

## 2. Materials and Methods

We conducted a population-based study among adult members of Leumit Health Services (LHS), a large, nation-wide Health Maintenance Organization (HMO) in Israel, which provides services to over 700,000 members. LHS has a comprehensive computerized database that is continuously updated regarding subjects’ demographics, medical diagnoses, medical encounters, hospitalizations and laboratory tests. Ethnicity was defined according to the home address of the HMO member and categorized into three groups: General population, Ultra-Orthodox Jews and Arabs. The latter two groups are of interest because a large-scale epidemiology study showed that they had significantly higher rates of infection than the rest of the Israeli population [9].

All LHS members have similar health insurance coverage and similar access to healthcare services. The validity of chronic diagnoses in the registry has been previously examined and confirmed as high [10,11].

Baseline data from individuals included in the cohort were extracted as of 15 November 2021, including age. All the clinical diagnoses were based on ICD-9 codes. During each physician visit, a diagnosis was entered or updated according to the International Classification of Diseases, 9th revision (ICD-9). We tested for the main medical conditions expected to affect the severity of COVID-19 infection in the adult population: diabetes mellitus, hypertension, asthma, chronic obstructive pulmonary disease, ischemic heart disease, presence of malignancy and chronic kidney disease.

### 2.1. SARS-CoV-2 Testing by Real-Time RT-PCR

Nasopharyngeal swabs were taken and examined for SARS-CoV-2 by real-time RT-PCR performed with internal positive and negative controls, according to World Health Organization guidelines. The Allplex 2019-nCoV assay (Seegene, Seoul, Korea) was used until 10 March 2020, after which time the COBAS SARS-Cov-2 6800/8800 assay (Roche Pharmaceuticals, Basel, Switzerland) was employed.

The study protocol was approved by the statutory research committee in LHS and the Shamir Medical Center Institutional Review Board on human research.

### 2.2. Assessment of Laboratory Parameters

In the study, the last blood analyses before booster vaccination were evaluated. All blood samples were collected <120 days before booster vaccination between 8 and 10 a.m. after a fasting night. Hematological and biochemical analyses of peripheral blood were performed at the LHS central laboratory.

### 2.3. Statistical Analyses

Standard descriptive statistics were used to present the demographic and clinical characteristics of patients included in this study. Differences in demographic, clinical and laboratory characteristics between groups were analyzed using independent sample *t*-tests for approximately normally-distributed continuous variables (described in tables by their mean and standard deviation) and Mann–Whitney U tests for other variables (described by their median and IQR). For categorical variables, proportions were tested using Fischer′s exact tests for binary variables and Chi Square tests for the age. Categorical data are shown in counts and percentages. Data on continuous variables are presented as mean and standard deviation; non-normal variables are displayed as median and interquartile range. All statistical analyses were conducted using R software version 4.0.2 (R Foundation).

### 2.4. Cohort Matching

Individuals with positive PCR results were matched to control individuals with negative PCR using a ratio of 1:5. Exact matching was required for sex, age category (marked by five years intervals), and demographic group (Jewish, Arab, and Jewish Ultra-Orthodox). Cases for which the required number of controls could not be found were not included in the matched cohort.

## 3. Results

Between 1 February 2021 and 30 November 2021, 136,683 individuals in LHS were vaccinated with the booster (third dose) of BNT162b2 vaccine. Of these, 1171 (0.9%) were diagnosed with COVID-19 by testing positive for SARS-CoV-2 RT-PCR at least >14 days after the booster vaccination. These individuals were infected with SARS-CoV-2 a mean of 81.08 days (SD 43.04) after their third vaccination and a mean of 304.79 days (SD 46.58) after their first vaccination (Table 1).

### 3.1. Demographic and Clinical Characteristics of Whole Vaccinated Population

The proportions of males and females in the COVID-19-positive and COVID-19-negative groups were equal.

1. The COVID-19-positive group was slightly younger (48.24 ± 17.63 years) than the COVID-19-negative group (49.54 ± 17.46 years; *p* = 0.011) and had significantly fewer smokers (77 (15.1%) vs. 20,201 (22.0%); *p* < 0.001) (Table 1).
2. There was no significant difference in the prevalence of several chronic comorbidities between the COVID-19-positive and COVID-19-negative groups, including arterial hypertension (311 (26.6%) vs. 38,669 (28.3%); *p* = 0.136), diabetes (184 (15.7%) vs. 21,228 (15.5%); *p* = 0.964), asthma (112 (9.6%) vs. 12,785 (9.4%); *p* = 0.879), COPD (56 (4.8%) vs. 7992 (5.8%); *p* = 0.106), ischemic heart disease (86 (7.3%) vs. 10,052 (7.4%); *p* = 0.496) and active malignancy (79 (6.7%) vs. 10,871 (8.0%); *p* = 0.109). The COVID-19-positive group was characterized by more cases of chronic kidney disease than the COVID-19-negative group (43 (3.7%) vs. 151 (2.6%); *p* = 0.037) (Table 2).

### 3.2. Demographic and Clinical Characteristics of the Study Population after Matching

Demographic and clinical characteristics of the study population after matching for sex, age and demographic factors (Jewish, Arab, Jewish Ultra-orthodox) in a 1:5 ratios are presented in Table 3. The clinical and laboratory parameters of the 1171 COVID-19 positive individuals were compared with 5855 COVID-19-negative cases.

1. The COVID-19-positive group was characterized by higher rates of chronic kidney disease than the matched COVID-19-negative group (43 (3.7%) vs. 3646 (2.7%); *p* = 0.039) (Table 2).
2. During the study period, 19 (1.6%) patients who were COVID-19 positive were hospitalized (7 (36.8%) with mild, 3 (15.8%) with moderate, and 5 (26.3%) with severe COVID-19), 2 (10.5%) patients were mechanically ventilated, and 4 (21.1%) patients died (Table 2). A multivariable logistic regression of the main parameters affecting COVID-19 positivity is provided as Supplementary Table S1.

### 3.3. Laboratory Characteristics of the Matched Population

Laboratory characteristics of the matched population are shown in Table 3.

There were very slight but significant differences in the various laboratory parameters between the matched groups.

1. In the COVID-19-positive group, hemoglobin, hematocrit, white blood cells, lymphocytes, monocytes, basophils, C3 Complement, total cholesterol, LDL cholesterol, prothrombin time (PT), thyroid stimulating hormone (TSH), free T4 and estimated glomerular filtration rate (eGFR) eGFR were lower.
2. Urea and albumin/creatinine ratio were higher in the COVID-19-positive group than in the COVID-19-negative group.

## 4. Discussion

In the study, we present data on BNT162b2 booster vaccination (third dose) of 136,683 community-based individuals in Israel, >14 days after their third BNT162b2 mRNA vaccination, with 1171 (0.9%) RT-PSR test-confirmed COVID-19 cases. We found a low rate of positive COVID-19 in those vaccinated with a booster dose, and anemia, lower WBC, lymphocytes, monocytes, basophils, and chronic kidney disease were associated with confirmed COVID-19 cases.

Despite the previously observed association between aging and a significant reduction in BNT162b2 mRNA vaccine-induced antibody responses after the first and second vaccinations, there remains a great need for studies focusing on people who are older [12,13]. The real-life study of adults older than 60 years demonstrated a significant immunogenicity after receiving the third dose of the BNT162b2 mRNA in all participants, even those who did not respond to previous doses [14]. However, increasing age is a recognized risk factor for COVID-19 and is also associated with more severe disease and poorer clinical outcomes [15]. The observed efficacy of the third BNT162b2 mRNA vaccination in people who are older suggests that the reduced immunogenicity observed after the first and second doses for this vaccine may be less relevant to the third booster vaccination.

Most of our individuals who were COVID-19 positive had asymptomatic or minimally symptomatic infection. Only 19 (1.6%) of them were hospitalized with a mortality rate of 21.1% (4 of 19), which is lower than the International Severe Acute Respiratory and Emerging Infection Consortium data (with a mortality rate of 27%) among persons hospitalized with COVID-19 in the United Kingdom after vaccination [16,17].

The reason for the association of lower hemoglobin and hematocrit with post-vaccination infection is unclear. Recently, Butt et al. reported that anemia was a significant risk factor for breakthrough SARS-CoV-2 infection after vaccination with two doses of the PfizerBNT-162b2 or Moderna-mRNA-1273 vaccines among US Veterans. The authors did not assess the association of the degree of anemia with the risk of SARS-CoV-2 infection [8]. Our study shows that a very mild and clinically insignificant drop in hemoglobin and hematocrit levels is already associated with the risk of SARS-CoV-2 infection after booster vaccination. Several research groups have previously reported the association between anemia in patients with COVID-19 infections and the associated risk of short-term mortality [18,19]. Remarkably, there was no difference in blood levels of iron, vitamin B12, or folic acid between our COVID-19-positive and COVID-19-negative groups. The observed lower hemoglobin levels are likely to reflect other underlying pathological conditions, often influenced by various concomitant diseases and/or risk factors. Therefore, we cannot say what potential pathophysiological mechanisms might be associated with SARS-CoV-2 infection after booster vaccination in our population.

Our study suggests that chronic kidney disease might be associated with a higher likelihood of SARS-CoV-2 infection after booster vaccination. This is noteworthy because individuals with chronic kidney disease were underrepresented in phase two and phase three studies of the COVID-19 vaccines [20]. This increased risk of SARS-CoV-2 infection after booster vaccination for people with chronic kidney disease could reflect impaired humoral immunity in these individuals [21].

In the last two years, several systematic reviews and meta-analyses have shown that the risk of SARS-CoV-2 infection is related to the burden of arterial hypertension and diabetes mellitus [22,23]. Nevertheless, our study did not identify hypertension and diabetes as two chronic diseases associated with a higher likelihood of SARS-CoV-2 infection after booster vaccination.

Patients with active cancer have a reduced humoral immune response and an increased risk of severe COVID-19 even after a double dose of BNT162b2 vaccination [24]. Nevertheless, the third dose of BNT162b2 can induce higher anti-SARS CoV-2 IgG titers than two doses in patients with active malignancy and immunosuppression [25]. Interestingly, we did not observe any association between active malignancy and COVID-19 after BNT162b2 booster vaccination.

The information on the presented laboratory variables distinguishing the COVID-19-positive from the COVID-19-negative group in the study must be interpreted with caution, as the small and clinically insignificant differences cannot be generalized. Nevertheless, these laboratory differences may be related to underlying alterations in cellular and humoral adaptive immune responses. Previous studies have focused on SARS-CoV-2 sequence data or the detection of antibodies in samples obtained from infected individuals following vaccination [26,27,28,29]. Therefore, the observed laboratory characteristics in individuals who are COVID-19 positive may be of interest to public health authorities, as there is an urgent need to understand the individual variables that are predisposed to breakthrough SARS-CoV-2 infection following booster vaccination in order to identify the population at risk.

Knowledge of the risk of SARS-CoV-2 infection after booster vaccination is essential in order to relax the sometimes very restrictive and psychologically stressful general lockdown measures and gradually return to pre-pandemic life. Therefore, our findings may have implications for strategies following booster vaccinations. Individuals who are fully vaccinated against COVID-19 may need to be cautious about physical distance and other personal protective measures in the post-vaccination period, especially if they have chronic kidney disease.

### 4.1. Study Strengths

Our study has several strengths. We studied an entire Israeli population with diverse geographic and demographic characteristics and cared for in a single health facility, and consulted a national database of individuals who have been SARS-CoV-2 RT-PCR screened and infected that uses validated definitions and algorithms, is regularly updated, and represents a productive source for clinical and observational studies. The design of our study, including matching cases and controls, reduced the potential for bias; however, small between-group differences in matched variables remained.

### 4.2. Study Limitations

Some limitations should be noted. We did not assess actual exposure to confirmed cases and were unable to rule out SARS-CoV-2 infection in asymptomatic individuals who were not tested using RT-PCR. Moreover, some of the factors that could affect infection risk were not included in the analysis, notably the occupation (i.e., healthcare workers could be at increased risk for developing SARS-CoV-2 infection), the characteristics of the household (i.e., individuals belonging to a household including more children may be at increased risk) and variation in the infectivity of viral variants. Another major limitation of the study is the lack of data on vaccine-specific antibody/T-cell response after booster vaccination. Precisely defined correlates of protection against SARS-CoV-2 infection have not yet been determined [30], neutralizing antibodies play an important role [31] and higher titers of neutralizing antibodies may be required to achieve cross-protection against SARS-CoV-2 variants after booster vaccination [32]. In addition, induction and boosting of S-specific T cells may play critical roles in the protection against SARS-CoV-2 [33]. S-specific T cells are capable of recognizing different SARS-CoV-2 variants, and thus, T-cell induction may be important in the face of declining antibody levels [34]. These crucial data would explain why patients had SARS-CoV-2 infection after the booster dose.

## 5. Conclusions

People with chronic kidney disease and anemia should be included in possible future annual SARS-CoV-2 vaccination recommendations.

## Figures and Tables

**Table 1:** Demographic and clinical characteristics of 136,683 subjects with Pfizer-BioNTech mRNA booster vaccination.

Number (%)	COVID-19 Positive	COVID-19 Negative	*p*
	1171 (0.86%)	135,512 (99%)	
Days since first vaccination; mean (SD)	304.8 (46.6)	294.71 (39.5)	<0.001
Days since second vaccination; mean (SD)	283.4 (46.6)	273.3 (39.7)	<0.001
Days since third vaccination; mean (SD)	81.1 (43.0)	69.1 (36.4)	<0.001
Gender, (female); *n* (%)	640 (55%)	70,406 (52%)	0.065
Age, Years; mean (SD)	48.2 (17.6)	49.5 (17.5)	0.011
19–29 Years; *n* (%)	145 (12%)	18,004 (13%)	<0.001
30–39 Years; *n* (%)	226 (19%)	19,594 (14%)	
40–49 Years; *n* (%)	207 (18%)	24,584 (18%)	
50–59 Years; *n* (%)	191 (16%)	26,297 (19%)	
60–69 Years; *n* (%)	214 (18%)	26,046 (19%)	
70–79 Years; *n* (%)	107 (9.1%)	12,650 (9.3%)	
80–89 Years; *n* (%)	29 (2.5%)	4490 (3.3%)	
≥90 Years; *n* (%)	7 (0.6%)	686 (0.5%)	
Jewish, *n* (%)	891 (76%)	109,548 (80%)	<0.001
Arab *n* (%)	119 (10%)	16,495 (12%)	0.036
Jewish Ultra-orthodox, *n* (%)	161 (14%)	10,645 (8%)	<0.001
Body mass index, (kg/m^2^) mean (SD)	26.7 (5.1)	27.1 (5.3)	0.010
Smoker, *n* (%)	147 (14%)	27,738 (22%)	<0.001
Comorbidity			
Diabetes mellitus, *n* (%)	184 (16%)	21,228 (16%)	0.964
Hypertension, *n* (%)	311 (27%)	38,669 (28%)	0.136
Asthma, *n* (%)	112 (9.6%)	12,785 (9.4%)	0.879
COPD, *n* (%)	56 (4.8%)	7992 (5.8%)	0.106
Ischemic heart disease, *n* (%)	86 (7.3%)	10,052 (7.4%)	0.496
Active malignancy, *n* (%)	79 (6.7%)	10,871 (8.0%)	0.109
Chronic kidney disease, *n* (%)	43 (3.7%)	3646 (2.7%)	0.039

**Table 2:** Demographic and clinical characteristics of the study population after matching.

	COVID-19 Positive	COVID-19 Negative	*p*
N	1171	5855	
Days since first vaccination; mean (SD)	304.8 (46.6)	303.7 (45.7)	0.439
Days since second vaccination; mean (SD)	283.4 (46.6)	282.3 (45.8)	0.426
Days since third vaccination; mean (SD)	81.1 (43.0)	77.6 (42.5)	0.010
Gender (female), *n* (%)	640 (55%)	3200 (55%)	0.999
Age, Years; mean (SD)	48.2 (17.6)	48.2 (17.5)	0.943
19–29 Years; *n* (%)	145 (12%)	725 (12%)	0.999
30–39 Years; *n* (%)	226 (19%)	1130 (19%)	
40–49 Years; *n* (%)	207 (18%)	1035 (18%)	
50–59 Years; *n* (%)	191 (16%)	955 (16%)	
60–69 Years; *n* (%)	214 (18%)	1070 (18%)	
70–79 Years; *n* (%)	107 (9.1%)	535 (9.1%)	
80–89 Years; *n* (%)	29 (2.5%)	145 (2.5%)	
≥90 Years; *n* (%)	7 (0.6%)	35 (0.6%)	
Jewish, *n* (%)	891 (76%)	4455 (76%)	0.999
Arab *n* (%)	119 (10%)	595 (10%)	0.999
Jewish Ultra-orthodox, *n* (%)	161 (14%)	805 (14%)	0.999
Body mass index (BMI), (kg/m^2^) mean (SD)	26.7 (5.1)	27.0 (5.3)	0.108
Smoker, *n* (%)	147 (14%)	1147 (21%)	<0.001
Comorbidity			
Hypertension, *n* (%)	311 (27%)	1521 (26%)	0.688
Diabetes mellitus, *n* (%)	184 (16%)	885 (15%)	0.593
Asthma, *n* (%)	112 (9.6%)	561 (9.6%)	0.999
COPD, *n* (%)	56 (4.8%)	304 (5.2%)	0.611
Ischemic heart disease, *n* (%)	86 (7.3%)	377 (6.4%)	0.272
Active malignancy, *n* (%)	79 (6.7%)	379 (6.5%)	0.745
Chronic kidney disease, *n* (%)	43 (3.7%)	151 (2.6%)	0.037
Hospitalization due to COVID-19	19 (1.6%)	0	<0.001
Mild	7 (37%)	0	
Moderate	3 (16%)	0	
Severe	5 (26%)	0	
Mechanical ventilation	2 (11%)	0	
Mortality since third vaccination, *n* (%)	4 (21%)	0	

**Table 3:** Laboratory characteristics of the matched population.

	COVID-19 Positive N = 1171	COVID-19 Negative N = 5855	*p*
Hemoglobin (g/dL); median [IQR]	13.60 [12.60–14.80]	13.80 [12.80–14.90]	0.022
RBC (10^6^/μL); median [IQR]	4.71 [4.36–5.09]	4.74 [4.39–5.10]	0.111
HCT (%); median [IQR]	40.70 [37.90–43.80]	41.00 [38.20–43.90]	0.028
MCV (fL); median [IQR]	86.70 [83.80–89.40]	86.90 [84.00–89.70]	0.135
WBC; median [IQR]	6.55 [5.59–7.84]	6.81 [5.70–8.09]	<0.001
Lymphocytes (10^9^/L); median [IQR]	2.05 [1.65–2.52]	2.14 [1.74–2.63]	<0.001
Lymphocytes % median [IQR]	31.70 [26.40–37.77]	32.30 [26.70–37.80]	0.191
Neutrophils (10^9^/L); median [IQR]	3.68 [2.92–4.52]	3.76 [2.97–4.75]	0.012
Neutrophils % median [IQR]	64.00 [47.00–74.65]	62.00 [46.00–72.20]	0.254
Monocytes (10^9^/L); median [IQR]	0.53 [0.36–0.78]	0.59 [0.37–0.89]	0.021
Monocytes % median [IQR]	8.00 [6.80–9.38]	7.90 [6.70–9.20]	0.115
Basophils (10^9^/L); median [IQR]	0.04 [0.03–0.06]	0.04 [0.03–0.06]	0.002
Basophils % median [IQR]	0.60 [0.40–0.80]	0.60 [0.40–0.80]	0.097
Eosinophils (10^9^/L); median [IQR]	0.16 [0.10–0.25]	0.17 [0.11–0.26]	0.065
Eosinophils %, median [IQR]	2.40 [1.70–3.80]	2.50 [1.70–3.70]	0.802
Platelets (10^9^/L); median [IQR]	241.00 [202.00–283.00]	242.00 [204.00–286.00]	0.355
MPV (fL); median [IQR]	87.30 [84.07–89.82]	86.90 [84.00–89.90]	0.515
Glucose (mg/dL); median [IQR]	96.45 [88.65–107.35]	96.20 [89.25–107.10]	0.911
Hemoglobin A1c (%); median [IQR]	5.50 [5.20–6.00]	5.50 [5.20–5.90]	0.932
Creatinine (mg/dL); median [IQR]	0.77 [0.64–0.94]	0.77 [0.65–0.92]	0.947
Urea (mg/dL); median [IQR]	30.20 [24.80–36.50]	29.45 [24.20–35.90]	0.039
Albumin/Creatinine Ratio	6.00 [3.56–15.10]	5.55 [3.29–12.81]	0.032
Iron (mcg/dL); median [IQR]	79.50 [62.65–101.35]	83.85 [62.40–105.38]	0.069
Ferritin (ng/mL); median [IQR]	62.69 [31.83–121.03]	67.73 [31.50–130.93]	0.198
Folic Acid (ng/mL); median [IQR]	7.10 [5.00–10.18]	7.02 [5.10–10.05]	0.762
C-Reactive Protein (mg/L); median [IQR]	2.40 [1.00–5.70]	2.80 [1.30–6.10]	0.020
ESR (mm/hr); median [IQR]	16.00 [10.00–29.00]	16.00 [9.00–28.25]	0.786
Total Protein (g/dL); median [IQR]	7.08 [6.76–7.33]	7.04 [6.78–7.33]	0.829
Albumin (g/dL); median [IQR]	4.20 [4.00–4.40]	4.20 [4.00–4.40]	0.252
25-OH Vitamin D (ng/mL); median [IQR]	22.10 [16.08–27.52]	22.40 [16.20–28.80]	0.317
Vitamin B12 (pg/mL); median [IQR]	372.20 [294.60–489.00]	379.95 [298.30–489.97]	0.399
Immunoglobulin A (mg/dL); median [IQR]	208.50 [156.50–292.75]	213.00 [153.00–289.25]	0.999
Immunoglobulin M (mg/dL); median [IQR]	107.00 [75.00–156.00]	108.00 [70.00–150.00]	0.745
Immunoglobulin G (mg/dL); median [IQR]	1141.00 [1033.00–1341.00]	1112.00 [963.00–1280.00]	0.102
Immunoglobulin E (IU/mL); median [IQR]	50.50 [13.88–111.25]	58.65 [18.00–200.25]	0.188
C3 Complement (mg/dL); median [IQR]	125.00 [113.00–139.00]	131.00 [115.00–148.00]	0.004
C4 Complement (mg/dL); median [IQR]	32.00 [26.50–38.00]	33.00 [28.00–40.00]	0.147
Total Cholesterol (mg/dL); median [IQR]	184.70 [158.43–215.25]	190.40 [161.97–217.93]	0.004
LDL Cholesterol (mg/dL); median [IQR]	111.00 [88.75–136.00]	115.00 [91.00–139.00]	0.004
HDL Cholesterol (mg/dL); median [IQR]	49.00 [41.00–57.00]	49.00 [42.00–58.00]	0.587
Triglycerides (mg/dL); median [IQR]	99.20 [70.50–141.30]	103.25 [74.40–146.62]	0.019
PT (INR); median [IQR]	0.98 [0.94–1.03]	0.97 [0.93–1.01]	0.004
PT (sec); median [IQR]	11.30 [10.70–11.90]	11.10 [10.50–11.60]	0.001
aPTT (sec); median [IQR]	28.60 [26.00–31.90]	27.80 [25.70–31.30]	0.059
TSH (mIU/L); median [IQR]	1.74 [1.19–2.45]	1.81 [1.27–2.51]	0.025
Free T4 (mIU/L); median [IQR]	13.02 [11.47–14.46]	13.23 [11.73–14.73]	0.021
Free T3 (mIU/L); median [IQR]	4.52 [4.05–4.96]	4.59 [4.12–5.04]	0.075
eGFR (mL/min/1.73 m^2^); mean (SD)	58.80 (5.62)	59.28 (4.24)	0.002
GFR G4/G5 < 15–29, *n* (%)	11 (0.9%)	33 (0.6%)	0.152
GFR G3b 30–44, *n* (%)	21 (1.8%)	56 (1.0%)	0.019
GFR G3a 45–59, *n* (%)	49 (4.2%)	197 (3.4%)	0.164
GFR G2 60–89, *n* (%)	362 (31%)	2019 (35%)	0.019
GFR G1 (Normal), *n* (%)	685 (59%)	3345 (57%)	0.401

Activated partial thromboplastin clotting time; TSH—Thyroid stimulating hormone; MPV—Mean platelet volume; ERS—Erythrocyte sedimentation rate; eGFR—Estimated glomerular filtration rate; PT—Prothrombin time; INR—International normalized ratio.

## Data Availability

This study is based on real-world patient data, including demographics and comorbidity factors, that cannot be communicated due to patient privacy concerns.

## Supplementary Materials

The following supporting information can be downloaded at https://www.mdpi.com/article/10.3390/vaccines10050636/s1, Table S1: Multivariable logistic regression of demographic and clinical characteristics affecting PCR positivity.

## References

[1] Raz A., Keshet Y., Popper-Giveon A., Karkabi M.S. (2021). One size does not fit all: Lessons from Israel’s Covid-19 vaccination drive and hesitancy. Vaccine.

[2] Covid-19 Dashboard [Internet]. https://datadashboard.health.gov.il/COVID-19/.

[3] Haas E.J., Angulo F.J., McLaughlin J.M., Anis E., Singer S.R., Khan F., Brooks N., Smaja M., Mircus G., Pan K. (2021). Impact and effectiveness of mRNA BNT162b2 vaccine against SARS-CoV-2 infections and COVID-19 cases, hospitalisations, and deaths following a nationwide vaccination campaign in Israel: An observational study using national surveillance data. Lancet.

[4] Israel A., Merzon E., Schäffer A.A., Shenhar Y., Green I., Golan-Cohen A., Ruppin E., Magen E., Vinker S. (2021). Elapsed time since BNT162b2 vaccine and risk of SARS-CoV-2 infection: Test negative design study. BMJ.

[5] Israel A., Shenhar Y., Green I., Merzon E., Golan-Cohen A., Schäffer A.A., Ruppin E., Vinker S., Magen E. (2021). Large-scale study of antibody titer decay following BNT162b2 mRNA vaccine or SARS-CoV-2 infection. Vaccines.

[6] Bar-On Y.M., Goldberg Y., Mandel M., Bodenheimer O., Freedman L., Kalkstein N., Mizrahi B., Alroy-Preis S., Ash N., Milo R. (2021). Protection of BNT162b2 Vaccine Booster against Covid-19 in Israel. N. Engl. J. Med..

[7] Barda N., Dagan N., Cohen C., Hernán M.A., Lipsitch M., Kohane I.S., Reis B.Y., Balicer R.D. (2021). Effectiveness of a third dose of the BNT162b2 mRNA COVID-19 vaccine for preventing severe outcomes in Israel: An observational study. Lancet.

[8] Butt A.A., Khan T., Yan P., Shaikh O.S., Omer S.B., Mayr F. (2021). Rate and risk factors for breakthrough SARS-CoV-2 infection after vaccination. J. Infect..

[9] Muhsen K., Na’Aminh W., Lapidot Y., Goren S., Amir Y., Perlman S., Green M.S., Chodick G., Cohen D. (2021). A nationwide analysis of population group differences in the COVID-19 epidemic in Israel, February 2020–February 2021. Lancet Reg. Health-Eur..

[10] Rennert G., Peterburg Y. (2001). Prevalence of selected chronic diseases in Israel. Isr. Med Assoc. J..

[11] Hamood R., Hamood H., Merhasin I., Keinan-Boker L. (2016). A feasibility study to assess the validity of administrative data sources and self-reported information of breast cancer survivors. Isr. J. Health Policy Res..

[12] Abu Jabal K., Ben-Amram H., Beiruti K., Batheesh Y., Sussan C., Zarka S., Edelstein M. (2021). Impact of age, ethnicity, sex and prior infection status on immunogenicity following a single dose of the BNT162b2 mRNA COVID-19 vaccine: Real-world evidence from healthcare workers, Israel, December 2020 to January 2021. Eurosurveillance.

[13] Yelin I., Katz R., Herzel E., Berman-Zilberstein T., Ben-Tov A., Kuint J., Gazit S., Patalon T., Chodick G., Kishony R. (2021). Associations of the BNT162b2 COVID-19 vaccine effectiveness with patient age and comorbidities. MedRxiv.

[14] Gilboa M., Mandelboim M., Indenbaum V., Lustig Y., Cohen C., Rahav G., Asraf K., Amit S., Jaber H., Nemet I. (2021). Early Immunogenicity and Safety of the Third Dose of BNT162b2 Messenger RNA Coronavirus Disease 2019 Vaccine Among Adults Older Than 60 Years: Real-World Experience. J. Infect. Dis..

[15] Dagan N., Barda N., Kepten E., Miron O., Perchik S., Katz M.A., Hernán M.A., Lipsitch M., Reis B., Balicer R.D. (2021). BNT162b2 mRNA Covid-19 Vaccine in a Nationwide Mass Vaccination Setting. N. Engl. J. Med..

[16] Docherty A.B., Mulholland R.H., Lone N.I., Cheyne C.P., De Angelis D., Diaz-Ordaz K., Donegan C., Drake T.M., Dunning J., Funk S. (2021). Changes in in-hospital mortality in the first wave of COVID-19: A multicentre prospective observational cohort study using the WHO Clinical Characterisation Protocol UK. Lancet Respir. Med..

[17] Egan C., Knight S., Baillie K., Harrison E., Docherty A., Semple C. (2021). Hospitalised Vaccinated Patients during the Second Wave. https://assets.publishing.service.gov.uk/government/uploads/system/uploads/attachment_data/file/982499/S1208_CO-CIN_report_on_impact_of_vaccination_Apr_21.pdf.

[18] Pilgram L., Eberwein L., Wille K., Koehler F.C., Stecher M., Rieg S., Kielstein J.T., Jakob C.E.M., Rüthrich M., The LEOSS Study Group (2021). Clinical course and predictive risk factors for fatal outcome of SARS-CoV-2 infection in patients with chronic kidney disease. Infection.

[19] Oh S.M., Skendelas J.P., Macdonald E., Bergamini M., Goel S., Choi J., Segal K.R., Vivek K., Nair S., Leff J. (2021). On-admission anemia predicts mortality in COVID-19 patients: A single center, retrospective cohort study. Am. J. Emerg. Med..

[20] Glenn D.A., Hegde A., Kotzen E., Walter E.B., Kshirsagar A.V., Falk R., Mottl A. (2021). Systematic Review of Safety and Efficacy of COVID-19 Vaccines in Patients With Kidney Disease. Kidney Int. Rep..

[21] Rincon-Arevalo H., Choi M., Stefanski A.-L., Halleck F., Weber U., Szelinski F., Jahrsdörfer B., Schrezenmeier H., Ludwig C., Sattler A. (2021). Impaired humoral immunity to SARS-CoV-2 BNT162b2 vaccine in kidney transplant recipients and dialysis patients. Sci. Immunol..

[22] Yang J., Zheng Y., Gou X., Pu K., Chen Z., Guo Q., Ji R., Wang H., Wang Y., Zhou Y. (2020). Prevalence of comorbidities and its effects in patients infected with SARS-CoV-2: A systematic review and meta-analysis. Int. J. Infect. Dis..

[23] Mattey-Mora P.P., Begle C.A., Owusu C.K., Chen C., Parker M.A. (2021). Hospitalised versus outpatient COVID-19 patients’ background characteristics and comorbidities: A systematic review and meta-analysis. Rev. Med Virol..

[24] Debie Y., Vandamme T., Goossens M.E., van Dam P.A., Peeters M. (2021). Antibody titres before and after a third dose of the SARS-CoV-2 BNT162b2 vaccine in patients with cancer. Eur. J. Cancer.

[25] Kamar N., Abravanel F., Marion O., Couat C., Izopet J., Del Bello A. (2021). Three Doses of an mRNA Covid-19 Vaccine in Solid-Organ Transplant Recipients. N. Engl. J. Med..

[26] Saha S., Al-Rifai R.H., Saha S. (2021). Diabetes prevalence and mortality in COVID-19 patients: A systematic review, meta-analysis, and meta-regression. J. Diabetes Metab. Disord..

[27] Philomina J.B., Jolly B., John N., Bhoyar R.C., Majeed N., Senthivel V., Cp F., Rophina M., Vasudevan B., Imran M. (2021). Genomic survey of SARS-CoV-2 vaccine breakthrough infections in healthcare workers from Kerala, India. J. Infect..

[28] Nixon D.F., Ndhlovu L.C. (2021). Vaccine Breakthrough Infections with SARS-CoV-2 Variants. N. Engl. J. Med..

[29] Jung J., Sung H., Kim S.H. (2021). Covid-19 Breakthrough Infections in Vaccinated Health Care Workers. N. Engl. J. Med..

[30] Krammer F. (2021). A correlate of protection for SARS-CoV-2 vaccines is urgently needed. Nat. Med..

[31] Khoury D.S., Cromer D., Reynaldi A., Schlub T.E., Wheatley A.K., Juno J.A., Subbarao K., Kent S.J., Triccas J.A., Davenport M.P. (2021). Neutralizing antibody levels are highly predictive of immune protection from symptomatic SARS-CoV-2 infection. Nat. Med..

[32] Planas D., Veyer D., Baidaliuk A., Staropoli I., Guivel-Benhassine F., Rajah M.M., Planchais C., Porrot F., Robillard N., Puech J. (2021). Reduced sensitivity of SARS-CoV-2 variant Delta to antibody neutralization. Nature.

[33] Tan A.T., Lim J.M., Le Bert N., Kunasegaran K., Chia A., Qui M.D., Tan N., Ni Chia W., de Alwis R., Ying D. (2021). Rapid measurement of SARS-CoV-2 spike T cells in whole blood from vaccinated and naturally infected individuals. J. Clin. Investig..

[34] Geers D., Shamier M.C., Bogers S., den Hartog G.D., Gommers L., Nieuwkoop N.N., Schmitz K.S., Rijsbergen L.C., van Osch J.A.T., Dijkhuizen E. (2021). SARS-CoV-2 variants of concern partially escape humoral but not T cell responses in COVID-19 convalescent donors and vaccine recipients. Sci. Immunol..

